# The prevalence of aberrant turbinates and nasal septum deviation in a Portuguese population of French bulldogs

**DOI:** 10.29374/2527-2179.bjvm006325

**Published:** 2025-11-12

**Authors:** Ana Carlos, Hugo Pereira, Alfredo Pereira, Maria Teresa Oliveira, David Ferreira

**Affiliations:** 1 Clínica Veterinária dos Milagres, Leiria, Portugal.; 2 Hospital Escolar da Faculdade de Medicina Veterinária, Universidade de Lisboa, Lisboa, Portugal.; 3 Mediterranean Institute for Agriculture, Environment and Development & Global Change and Sustainability Institute, Departamento de Zootecnia, Universidade de Évora, Escola de Ciências e Tecnologia, Universidade de Évora, Évora, Portugal.; 4 CHRC – Comprehensive Health Research Centre, Departamento de Medicina Veterinária, Escola de Ciências e Tecnologia, Universidade de Évora, Évora, Portugal.

**Keywords:** brachycephalic obstructive airway syndrome, aberrant turbinates, nasal septum deviation, French bulldog, computed tomography, síndrome das vias aéreas obstrutivas braquicefálicas, turbinados aberrantes, desvio do septo nasal, buldogue francês, tomografia computorizada

## Abstract

The presence of nasal septum deviation (NSD) and aberrant turbinates alters normal nasal airflow. The role of NSD in brachycephalic obstructive airway syndrome (BOAS) is unclear but, together with other anatomical alterations such as aberrant turbinates, may alter nasal airflow, increasing upper airway resistance. This retrospective study reports the prevalence of rostral (RAT) and caudal (CAT) aberrant turbinates, and NSD in a population of 45 French Bulldogs without clinical manifestations of upper airway obstruction who underwent head computed tomography (CT) for reasons unrelated to BOAS. Forty-five French Bulldogs were studied, and NSD, RAT, and CAT were identified in 69%, 44%, and 64% of cases, respectively. Animals weighing 11 - 14 kg were more likely to have NSD than animals weighing 8 - 11 kg. Conversely, animals weighing 8 – 11 kg were more likely to present CAT than animals weighing 11 - 14 kg, with a marginal statistical significance. No correlations were observed between body weight and RAT, nor between body weight and gender or reproductive status. The higher frequency of NSD-affected French Bulldogs observed in our study, when compared to previous studies in French Bulldogs with BOAS, suggests that NSD may play a minor role in upper airway obstruction within the entire bundle of anatomical aberrations present in the skull of French Bulldogs. On the other hand, RAT may play a more significant role in the development of clinical manifestations of upper airway obstruction.

## Resumo

A presença de desvio do septo nasal (NSD) e de turbinados aberrantes (AT) altera o fluxo aéreo nasal normal. A relevância do NSD na síndrome braquicefálica (BOAS) não é clara, mas, em conjunto com outras alterações anatómicas, como os AT, pode interferir no fluxo aéreo, aumentando a resistência das vias aéreas superiores. O presente estudo retrospetivo relata a prevalência de AT rostrais (RAT), caudais (CAT) e de NSD numa população de 45 Buldogues Franceses sem manifestações clínicas de obstrução das vias aéreas superiores, submetidos a tomografia computorizada da cabeça por motivos não relacionados com BOAS. Foram estudados 45 animais e os NSD, RAT e CAT estiveram presentes em 69%, 44% e 64% dos casos, respetivamente. Animais com peso entre 11 e 14 kg têm maior probabilidade de ter NSD do que animais com peso entre 8 e 11 kg. Animais com peso entre 8 e 11 kg são mais propensos a apresentar CAT do que animais com peso entre 11 e 14 kg, com uma significância marginal. Não foram observadas correlações entre peso e RAT, nem entre peso e género ou estado reprodutivo. A presente observação no nosso estudo da elevada percentagem de Buldogues Franceses afetados por NSD, quando comparada com estudos anteriores realizados em Buldogues Franceses com BOAS sugere que, dentro do feixe de aberrações anatómicas no crânio de buldogues franceses, o NSD pode ter uma menor importância no desenvolvimento de manifestações clínicas associadas à obstrução das vias aéreas superiores.

## Introduction

Brachycephalic syndrome, brachycephalic respiratory syndrome, or brachycephalic obstructive airway syndrome (BOAS) is a complex hereditary disorder (Ekenstedt et al., 2020) that induces, among others, severe respiratory distress in brachycephalic breeds (Meola, 2013). These respiratory and consequent thermoregulation problems result from congenital anatomical alterations causing a partial or complete obstruction of the upper airways (Auger et al., 2016; Ekenstedt et al., 2020; Oechtering et al, 2016b). The symptoms associated with BOAS tend to worsen when the animal is obese, in situations of excitement, stress, exercise, or at increases in environmental temperature or humidity (Auger et al., 2016; Bofan et al., 2015; Ekenstedt et al., 2020; Meola, 2013; Oechtering et al., 2016b).

BOAS is characterized by several primary anatomical alterations such as: i) stenosis of the nares and nasal vestibule (Auger et al., 2016; Bofan et al., 2015; Ekenstedt et al., 2020; Meola, 2013; Oechtering et al., 2016a; Oechtering et al., 2016b); ii) rostral aberrant turbinates (RAT); iii) caudal aberrant turbinates (CAT) (Miles & Schwarz, 2020; Vilaplana Grosso et al., 2015) causing turbulent airflow and inspiratory resistance (Auger et al., 2016); iv) macroglossia (Heidenreich et al., 2016; Koch et al., 2003; Meola, 2013; Oechtering et al., 2016b; Pichetto et al., 2011); and v) tracheal hypoplasia (Davis et al., 2017; Kaye et al., 2018; Meola, 2013; Oechtering et al., 2016b; Schuenemann et al., 2017). There are also secondary sequelae such as: i) everted laryngeal ventricles; ii) laryngeal collapse, present in 53% of dogs with BOAS, especially in those with advanced states of this syndrome; iii) tracheal collapse; iv) bronchial collapse; v) soft palate hyperplasia; and vi) excessive aryepiglottic folds (Bofan et al., 2015; MacPhail, 2020; Meola, 2013; Pink et al., 2006). These secondary sequelae lead to the exacerbation of the BOAS clinical signs and may, in extreme cases, cause the animals’ death (Bofan et al., 2015; Heidenreich et al., 2016; Meola, 2013).

Computed tomography scans in dogs with BOAS have revealed that the shortening of the skull in brachycephalic breeds also causes the protrusion of ectopic tissue into the nasal cavity, because it cannot be accommodated by the internal head structures due to the shortening of the skull (Liu et al., 2017; Oechtering et al., 2007; Oshita et al., 2022). Reduced nasal cavity, soft tissue hypertrophy, and congenital NSD contribute to increasing airway resistance. Intranasal mucosa contact points can also be considered an acquired sequela in dogs suffering from BOAS, since nasal turbinates continue to grow after birth (Vilaplana Grosso et al., 2015).

A recent prospective clinical study by Oechtering et al. (2016a) reported that 66.7% of brachycephalic dogs had aberrant nasal turbinates, and 91.7% of the dogs had interconchal and intraconchal mucosal contacts (Oechtering et al., 2016a). According to the same authors, the Pug is the breed with the highest percentage of aberrant nasal turbinates (RAT= 90.9% and CAT= 69.7%), followed by French Bulldogs (RAT= 56.4% and CAT= 65.5%) and English Bulldogs (RAT= 36.4% and CAT= 54.5%) (Oechtering et al., 2016a).

To better understand the contribution of anatomical alterations in the nasal cavity of French Bulldogs in the development of clinical manifestations of upper airway obstruction, it is important to study the prevalence of these aberrations in a population of animals that do not show clinical signs of upper airway obstruction. The present retrospective study reports the prevalence of RAT, CAT, and NSD in French Bulldogs without clinical manifestations of upper airway obstruction that, for reasons unrelated to BOAS, were submitted to computed tomography exams of the head during a five-year period (July 2015 – July 2020).

## Materials and methods

### Inclusion criteria

The inclusion criteria of the study were the following: a) French Bulldogs; b) over one year of age; c) males and females regardless of the reproductive status; d) submitted to head computed tomography scans for reasons unrelated to BOAS in a Portuguese veterinary hospital (Hospital Veterinário do Restelo, HVR), Lisbon, Portugal, during a five-year period (July 2015 – July 2020).

### Database and research methodology

The entire data were selected from the HVR clinical archive using the veterinary clinical software QVET© (Q-SOFT, Lleida, Spain) with the scrupulous application of the Portuguese Law 58/2019 for the Protection of Personal Data [22]. Data was searched using the filters "Reproductive status: all", "Species: canine", "Breed: French Bulldog", "Dates: 01-06-2015 to 01-06-2020 period" and "Type of consultation: tomography", and information about sex, reproductive status, age, body weight, and computed tomography images were collected from each animal that appeared in the search.

### Computed tomography images examination

Computed tomography images were acquired with two scanners: HiSPeed LX/I (GE Healthcare, Chicago, USA; from July 2015 to September 2016), and SOMATOM Emotion 16 (Siemens Healthcare, Erlangen, Germany; from October 2016 to June 2020). The scans were performed in French Bulldogs that were anesthetized according to each individual need. Ranitidine 2 mg/kg IV (Bloculcer 50mg/2ml, Labesfal, Santiago de Besteiros, Portugal), maropitant 2 mg/kg SC (Cerenia 10 mg/ml, Zoetis, Louvain-la-Neuve, Belgium) and metoclopramide 2 mg/kg IV (Metoclopramida Labesfal, 10 mg/2ml, Labesfal, Santiago de Besteiros, Portugal) were administered to all animals prior to anesthesia. Anesthetic premedication included fentanyl 2 mcg/kg IV (Fentadon 50 mcg/ml, Dechra, Barcelona, Spain) and midazolam 0.2 mg/kg IV (Midazolam Labesfal 15 mg/3ml, Labesfal, Santiago de Besteiros, Portugal), and anesthetic induction was performed using propofol 2 mg/kg IV (Propofol-Lipuro 10 mg/ml (1%) VET, B. Braun, Melsungen, Germany). All animals were intubated, and maintenance of anesthesia was with isoflurane (IsoVet 1000 mg/g, B. Braun, Melsungen, Germany) volatilized in a mixture of air and oxygen.

After induction of anesthesia, the anesthetized dogs were positioned on the computed tomography table in a symmetrical supine position, with the neck and head extended position. During the computed tomography scan, the animals were monitored by continuous ECG and pulse oximetry. Computed tomography scans were performed from the nasal plane to the level of the axis (cranial CT), and tomographic images were obtained using a bone algorithm with a thickness of cut/sections of 0.75 mm. After obtaining the series of transverse images, these were used for multiplanar reformatting of the dorsal, transverse, and sagittal planes. All the computed tomography scans morphometric analyses were performed by the same technician, using the software Horos™ Version 3 (LGPL-3.0). All images were analyzed in the transverse, dorsal, and sagittal planes.

Nasal cavity images were examined for NSD and abnormal growth of the nasal turbinates. The dorsal, transverse, and sagittal turbinates were analyzed for the presence or absence of RAT, CAT, and NSD. Images in transverse planes were used for identifying RAT, while images in the sagittal plane were used for identifying CAT, the dorsal and transverse planes were used for identifying NSD. For the purposes of this study, CAT were considered when caudal nasal turbinates extended caudally from the nasal choana into the nasopharynx opening, passing ventrally to the wings of the vomer; RAT were considered when the laminae of middle nasal or of the ventral nasal turbinate protruded rostrally to the point of the first branch of the alar fold, obstructing the common and middle nasal meatus [7].

### Data analysis

The collected data were organized using Microsoft Office Excel® (Microsoft Office 2019, Microsoft Corporation, Washington, USA). For each animal, the following variables were collected: gender, reproductive status, age, body weight and the presence (marked as "1") or absence (marked as "0") of RAT, CAT or NSD.

Results were analyzed using SPSS Statistics 28 (IBM Corp., Armonk, NY, USA). Data are presented as means ± standard deviations (SD). Fisher's test was used to explore the existence of an association between the reproductive state of the animals and the NSD, RAT, and CAT, and between NSD, RAT, and CAT. The point-biserial correlation coefficient was used to explore the correlation between age and weight, and the NSD, RAT, and CAT. The Chi-square test was also used to compare the variables NSD, RAT, and CAT, with body weight and age, and to evaluate the frequency of NSD, RAT, and CAT distribution in the present population of French Bulldogs. Also, the correlation between weight and sex (regardless of reproductive status), and between body weight and desexed status was performed using Spearman’s Rank correlation. The results were considered statistically significant with a p<0.05.

## Results

### Characterization of the population

Fifty-six animals were selected from the database for meeting the search criteria. From these, eleven animals were excluded from data analyses due to chronic nasal disease (one animal) and damaged computed tomography files that did not allow image analysis (10 animals). Thus, a total of 45 French Bulldogs were included for data analyses. The demographic data are represented in Table 1.

**Table 1:** Demographic data from the 45 French Bulldogs included in the study.

**Animals**	**n**	**% 1**	**% 2**	**Age range (years)**	**Age (years) (Mean ± SD)**	**BW (Kg) (Mean ± SD)**
NM	6	13.3%	25%	1 - 7	3.8 ± 2.0	13.3 ± 2.8
IM	18	40%	75%	1 - 9	4.8 ± 2.4	14.5 ± 2.7
Total Males	24	53.3%	100%	1 - 9	4.6 ± 2.3	14.2 ± 2.7
SF	14	31,1%	66,7%	1 - 13	5.9 ± 3.7	11.0 ± 2.2
IF	7	15,6%	33,3%	1 - 8	2.9 ± 2.5	12.0 ± 3.8
Total Females	21	46.7%	100%	1 - 13	4.9 ± 3.6	11.3 ± 2.8

^1%^ for the entire sample;

^2%^ for the group of males or females;

BW – Body Weight; SD – Standard Deviation; NM – neutered male; IM – intact male; SF – spayed female; IF – intact female.

In the population analyzed, 2% of the French Bulldogs (1/45) did not present RAT, CAT, or NSD; 33% (15/45) presented one single anatomical alteration; 49% (22/45) presented two anatomical alterations; and 16% (7/45) presented three anatomical alterations.

### Computed Tomography Examination

**Figure 1:**
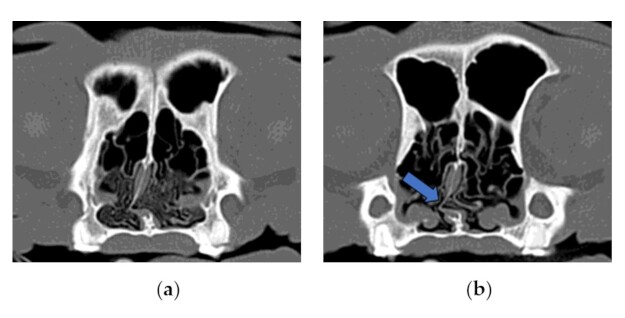
Transverse computed tomography scans of the nasal cavity of a French Bulldog of the study, a) at the level of the infraorbital foramen, and b) at the level of 4th premolar teeth. The arrow in image b) points to the nasal septum deviation.

**Figure 2:**
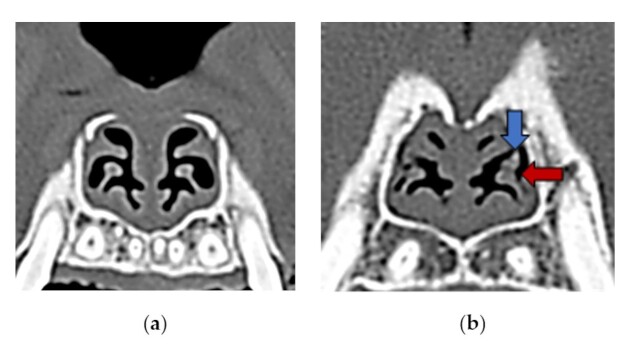
Transverse computed tomography scans of the rostral nasal cavity of a French Bulldog of the study. (a) Absence of rostral aberrant turbinates; (b) Presence of rostral aberrant turbinates (blue arrow) branching rostrally to the first branch of the alar fold (red arrow).

**Figure 3:**
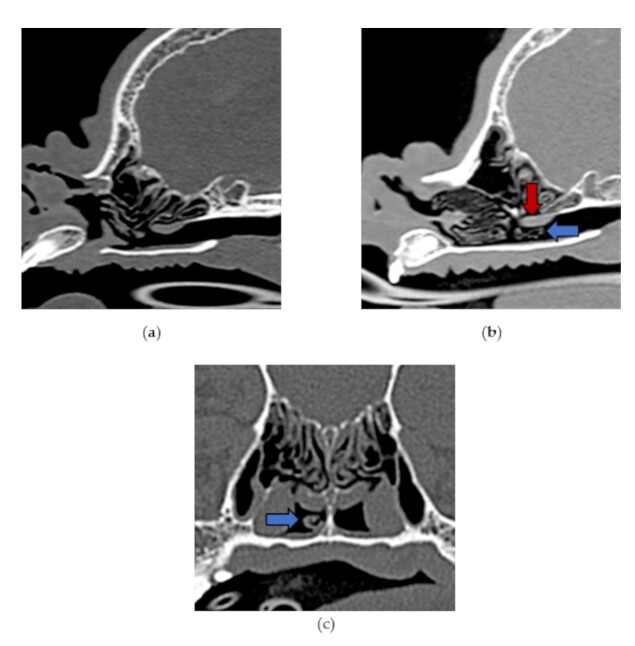
Sagittal ((a) and (b)) and transverse (c) computed tomography scans of the nasal cavity of a French Bulldog of the study. (a): Absence of caudal aberrant turbinate (b): Presence of caudal aberrant turbinate caudally projecting into the nasopharyngeal meatus (blue arrow) and passing ventrally to the wing of the vomer (red arrow); (c): Presence of caudal aberrant turbinates (blue arrow) at the nasopharyngeal meatus.

Computed tomography scans were analyzed for NSD, RAT, and CAT. Figures 1, 2 and 3 were obtained from dogs included in our study, and show, respectively, an example of NSD, RAT, and CAT.

### Population frequency of NSD, RAT and CAT

The analysis of computed tomography scans revealed that 44.4% (n=20) of the animals had RAT, 64.4% (n=29) had CAT and 68.9% (n=31) had NSD. Detailed data are presented in Table 2.

**Table 2:** Descriptive analysis of the population regarding anatomical anomalies (RAT, CAT and NSD).

**Animals**	**RAT (n)**	**% RAT 1**	**CAT (n)**	**% CAT^1^**	**NSD (n)**	**% NSD^1^**
NM	2/6	33,3%	4/6	66.7%	5/6	83.3%
uIM	8/18	44,4%	13/18	72.2%	12/18	66.7%
Total Males	10/24	41.6%	17/24	70.1%	17/24	70.1%
SF	7/14	50%	8/14	57.1%	9/14	64.3%
IF	3/7	42,9%	4/7	57.1%	5/7	71.4%
Total Females	10/21	47.6%	12/21	57.1%	14/21	66.7%

^1%^ relative percentage;

RAT – Rostral aberrant turbinates; CAT – caudal aberrant turbinates; NSD – nasal septum deviation; NM – neutered male; IM – intact male; SF – spayed female; IF – intact female.

### Frequency of the distribution of NSD, RAT and CAT

The NSD is a frequent characteristic in the population of French Bulldogs in our study, with about 70% of the animals presenting this anomaly (p= 0.0112). Also, although with a marginal significance, the authors can say with some degree of certainty that it is expected that 64% of the French Bulldogs present CAT (p= 0.0562). On the other hand, the relative RAT frequency observed in our study was approximately 44% (p= 0.4561).

### Association between the reproductive status of the animals and NSD, RAT and CAT, and between the variables NSD, RAT and CAT

There was no association between the reproductive status and the presence of NSD (p=0.9325), RAT (p=0.971) or CAT (p=0.843). Also, no association was observed between NSD and RAT (p=0.7488), NSD and CAT (p=0.7378), or between RAT and CAT (p=0.3484). There was no correlation between gender or reproductive status and body weight.

### Correlation between age and body weight, and the presence of NSD, RAT and CAT

There was no correlation between age and weight, and the presence of NSD, RAT and CAT (Table 3).

**Table 3:** Correlation analysis between age and BW, and the presence of NSD, RAT and CAT.

**Variables**	**Age**		**BW**	
	**Correlation coefficient (r-value)**	**Significance (p-value)**	**Correlation coefficient (r-value)**	**Significance (p-value)**
NSD	0.03	0.8326	0.06	0.6880
RAT	0.07	0.6300	0.12	0.4402
CAT	0.17	0.2633	-0.03	0.8290

BW – body weight; NSD – nasal septum deviation; RAT – Rostral aberrant turbinates; CAT – caudal aberrant turbinates.

On the other hand, animals weighing between 11 and 14 kg are more likely to have NSD than animals weighing between 8 and 11 kg (p=0.0254). Animals weighing between 8 and 11 kg are more likely to present CAT than animals weighing between 11 and 14 kg, with marginal significance (p= 0.0508). The weight groups were defined by calculating the median and quartiles of the sample, which made it possible to put the weights of the individuals into five classes.

## Discussion

A correct diagnosis of BOAS is obtained based on breed, medical history, clinical presentation of the animal and on visual examination of the nostrils (awake animal), palate and larynx (under sedation) (Bofan et al., 2015; Heidenreich et al., 2016; Meola, 2013). Complementary diagnostic tests such as cervical and thoracic radiographs, computed tomography scans to the head, endoscopic examination of the upper airways and gastrointestinal tract, and functional testing are also essential for a complete BOAS diagnosis (Heidenreich et al., 2016). Whole‐body barometric plethysmography and impulse oscillometry also provide useful information regarding the airflow and intranasal resistance in brachycephalic dogs but are impractical for clinical use (De Lorenzi et al., 2009). A German survey involving owners of 37 Pugs and 25 French Bulldogs that needed surgical intervention, reported that 95% of the animals had exercise intolerance (75% of whom could only walk for 10-30 minutes), 68% had sleep disorders (30% had apnea or tried to sleep sitting), and that 77% of the French Bulldogs had gastrointestinal disorders (Schuenemann et al., 2017). These gastrointestinal disorders included dysphagia, ptyalism, regurgitation, and vomiting, that may be associated with the presence of hernia of the hiatus, pyloric stenosis, esophageal deviation, esophageal diverticulum, or reflux esophagitis (Ekenstedt et al., 2020; Heidenreich et al., 2016; Kaye et al., 2015; Roedler et al., 2013; Scales & Clancy, 2019a; Schuenemann et al., 2017). Regurgitation may result in aspiration pneumonia, thus increasing the risk of pulmonary fatigue (Scales & Clancy, 2019b). The French Bulldogs included in our retrospective study were not submitted to a thorough BOAS diagnostic approach and, although they did not present clinical manifestations of upper airway obstruction at examination prior to the head computed tomography scan, they cannot be considered BOAS free. Our study evaluated the prevalence of anatomical aberrations in the nasal cavity of French Bulldogs that, although present, were not causing clinical manifestations of upper airway obstruction at the time of the study.

Computed tomography was established as a reference diagnostic tool for nasal disease in dogs, and its routine use is leading to a better understanding of nasal diseases in brachycephalic dogs (Miles & Schwarz, 2020). Although the bibliography with detailed computed tomography information on nasal morphological impairment in brachycephalic dogs is still scarce (Heidenreich et al., 2016; Oechtering et al., 2007; Oshita et al., 2022), it reports a high prevalence of RAT, CAT, and NSD in brachycephalic dogs with BOAS (Liu et al., 2019; Oechtering et al., 2007; Oechtering et al., 2016a).

The prevalence of aberrant turbinates in French Bulldogs with severe BOAS has been reported to be 65.5% for CAT (Oechtering et al., 2016a). These results are similar to the 64% observed in our study. These similar CAT percentages, observed in French Bulldogs with or without clinical manifestations of upper airway obstruction suggest that CAT does not play an important individual role in the manifestation of BOAS in French Bulldogs. On the other hand, the prevalence of RAT in French Bulldogs with severe BOAS is reported to be 55.6% (Liu et al., 2019) or 56.5% (Oechtering et al., 2016a), while the prevalence of RAT observed in the French Bulldogs in our study was 44.4%. This approximately 10-12% difference between these published studies and our study suggests that RAT themselves may play an active role in upper airway obstruction. It is logical to assume that the short and narrower lumen of the rostral nasal cavity may more easily be obstructed by RAT, compared to CAT in the wider caudal nasal cavity. Nevertheless, and despite these observations, the level of the turbinates’ deformities is a factor to be considered.

The presence of NSD in brachycephalic dogs was first described by Zinram (1933), who associated the high occurrence of NSD with the reduction of the viscerocranium (Zinram, 1933). More recent studies have reported that NSD is a common anatomical deformity in any type of dog. However, the degree of deviation is directly proportional to the cranial index (Miles & Schwarz, 2020; Oshita et al., 2022). The high cranial indices characteristic of brachycephaly are associated with shorter NSDs with higher angulation, because the shorter the viscerocranium, the smaller the expansion of the NSD in length (Miles & Schwarz, 2020).

Oechtering and colleagues reported a NSD prevalence of 14.5% in a population of 55 French Bulldogs with a mean body weight of 11.9 ± 2.3 kg, referred for surgical approach to severe BOAS clinical signs (Oechtering et al., 2016a). On the other hand, Schuenemann and colleagues reported a NSD prevalence of 27.5% in a population of 40 French Bulldogs with mean body weight of 11.4 ± 2.3kg, also referred for surgical treatment of clinical signs related to severe BOAS (Schuenemann et al., 2017). In our study, the NSD prevalence of 68.9% was much higher than that reported (Oechtering et al., 2016a; Schuenemann et al., 2017). Nevertheless, the NSD was not causing clinical manifestations of BOAS in the animals in our study.

Also, it was observed in our study that French Bulldogs weighing between 11 and 14 kg were more likely to have NSD than French Bulldogs weighing between 8 and 11 kg. On the other hand, French Bulldogs weighing between 8 and 11 kg were more likely to have CAT than French Bulldogs weighing between 11 and 14 kg. These observations can be justified by the genetic component of brachycephalic breeds (characterized by high cranial indices/a reduced viscerocranium) (Selba et al., 2020; Vilaplana Grosso et al., 2015), as well as by the extrinsic forces (Vilaplana Grosso et al., 2015) and tensions/torsions exerted on the fetal skull during pregnancy (Neskey et al., 2009) and/or delivery, or other lesions (Vilaplana Grosso et al., 2015).

The results from our study suggest that it is essential to further study the anatomical alterations of the nasal cavity of French Bulldogs and its relationship with clinical manifestations of upper airway obstruction.

## Conclusions

The higher NSD prevalence observed in the French Bulldogs included in our study, when compared to other published studies in French Bulldogs with BOAS, suggests that NSD plays a minor role in the development of clinical manifestations of upper airway obstruction. On the other hand, RAT may play a relevant role in the development of clinical manifestations of upper airway obstruction as suggested by the observed lower percentage of French Bulldogs with RAT in our study, compared to French Bulldogs with severe BOAS included in other published studies.
