# ERRATUM: “The prevalence of aberrant turbinates and nasal septum deviation in a Portuguese population of French bulldogs”

**DOI:** 10.29374/2527-2179.bjvm003326

**Published:** 2026-03-12

Due to an omission by the authors, the article “The prevalence of aberrant turbinates and nasal septum deviation in a Portuguese population of French bulldogs” (DOI https://doi.org/10.29374/2527-2179.bjvm006325), published in the *Brazilian Journal of Veterinary Medicine*, 47(1): e006325, was published with an error in funding information.

On page 8, below the “Financial support” section, it is shown as:

None.

It should show:

“This work is funded by national funds through the Foundation for Science and Technology, under the project UID/06291/2025”

The authors apologize for this error.

